# Alveolar Macrophages: Adaptation to Their Anatomic Niche during and after Inflammation

**DOI:** 10.3390/cells10102720

**Published:** 2021-10-12

**Authors:** Florian Pierre Martin, Cédric Jacqueline, Jeremie Poschmann, Antoine Roquilly

**Affiliations:** 1 EA3826 Host Pathogen Interactions, Inflammation and Mucosal Immunity, Department of Anesthesiology and Intensive Medicine, Hôtel Dieu, CHU Nantes, University of Nantes, F-44000 Nantes, France; florian.martin2@etu.univ-nantes.fr (F.P.M.); cedric.jacqueline@univ-nantes.fr (C.J.); 2 Centre de Recherche en Transplantation et Immunologie, University of Nantes, UMR 1064, ITUN, Inserm, F-44000 Nantes, France; jeremie.poschmann@univ-nantes.fr

**Keywords:** resident alveolar macrophages, lung microenvironment, alveolar niche, trained immunity, pneumonia, inflammatory monocytes

## Abstract

At the early stages of life development, alveoli are colonized by embryonic macrophages, which become resident alveolar macrophages (ResAM) and self-sustain by local division. Genetic and epigenetic signatures and, to some extent, the functions of ResAM are dictated by the lung microenvironment, which uses cytokines, ligand-receptor interactions, and stroma cells to orchestrate lung homeostasis. In resting conditions, the lung microenvironment induces in ResAM a tolerogenic programming that prevents unnecessary and potentially harmful inflammation responses to the foreign bodies, which continuously challenge the airways. Throughout life, any episode of acute inflammation, pneumonia being likely the most frequent cause, depletes the pool of ResAM, leaving space for the recruitment of inflammatory monocytes that locally develop in monocyte-derived alveolar macrophages (InfAM). During lung infection, the local microenvironment induces a temporary inflammatory signature to the recruited InfAM to handle the tissue injury and eliminate the pathogens. After a few days, the recruited InfAM, which locally self-sustain and develop as new ResAM, gain profibrotic functions required for tissue healing. After the complete resolution of the infectious episode, the functional programming of both embryonic and monocyte-derived ResAM remains altered for months and possibly for the entire life. Adult lungs thus contain a wide diversity of ResAM since every infection brings new waves of InfAM which fill the room left open by the inflammatory process. The memory of these innate cells called trained immunity constitutes an immunologic scar left by inflammation, notably pneumonia. This memory of ResAM has advantages and drawbacks. In some cases, lung-trained immunity offers better defense capacities against autoimmune disorders and the long-term risk of infection. At the opposite, it can perpetuate a harmful process and lead to a pathological state, as is the case among critically ill patients who have immune paralysis and are highly susceptible to hospital-acquired pneumonia and acute respiratory distress syndrome. The progress in understanding the kinetics of response of alveolar macrophages (AM) to lung inflammation is paving the way to new treatments of pneumonia and lung inflammatory process.

## 1. Introduction

Historically, AM were defined by their monocytic origin and high phagocytic functions against pathogens colonizing sterile lungs. The last decades swept these old dogmas away by showing that embryonic macrophages colonize the lung during the first week of life in mice [1] and are in charge of the lung tissue homeostasis [2], where a microbiota naturally dwells [3]. AM play a significant role in pulmonary physiology as some alterations of their functions can lead to severe or even lethal respiratory diseases [4]. Macrophages are also classically separated into two subpopulations: one oriented towards inflammation and antimicrobial defense known as classically activated macrophages (M1), opposing the one oriented towards immune tolerance and tissue repair known as alternatively activated macrophages (M2) [5]. This theoretical dichotomy was recently challenged with in vivo data showing the coexistence of M1 and M2 features in the same macrophages depending on the experimental conditions [6], thus limiting the M1/M2 classification to in vitro models [7]. In the present review, we have summarized the recent data about the ontogeny of AM that colonize the alveolar niche at birth and during inflammation. Then we focused on maintaining immune tolerance by the regulatory interactions between the lung microenvironment and the AM. We then have related the evolution of the alveolar niche following lung inflammation, from the initial ResAM elimination to the InfAM recruitment. The difference of functions between these two cell populations raises the question of the organization of their coexistence in the niche. We then discussed the roles of the ResAM and InfAM during lung inflammation and its resolution. Finally, we described the immunological scar left over by inflammation, also known as trained immunity, its long-term implication, between advantage and drawback.

## 2. Resident Alveolar Macrophages Ontogeny

Contrary to the former dogma stipulating that all macrophages originated from circulating bone marrow-derived monocytes, ResAM are known for their embryogenic origin, which requires a small contribution from monocytic progenitors during homeostasis. The alveolar colonization by the ResAM occurs during the first week of life in mice, and experiments using irradiated chimeric mice, parabiotic mice, and adoptive cell transfer showed the alveolar colonization was dependent on specific embryonic progenitors [1,8,9]. Yolk sac derived-macrophages, fetal liver monocytes, or bone marrow-derived monocytes can also acquire efficient macrophage functions, such as preventing proteinosis and phagocytosis. Their contribution to the final ResAM pool is not equivalent, the fetal liver monocytes constituting the main proportion of resident macrophages. Many studies that have looked at other tissue-specific macrophages obtained different results, which supposes that every type of macrophage gets its specific preferential embryonic origin. Tissue-specific macrophages from the microglia [10,11], the liver, the spleen, and the pancreas originate mainly from the yolk sac [12], while intestinal macrophages are constantly replaced by bone marrow-derived monocytes [13]. The contribution from different sources of embryonic progenitors varies in a tissue-dependent manner, and the mechanisms of this tissue imprinting are starting to be elucidated.

## 3. Maintenance of the Alveolar Macrophage Niche during Homeostasis

The observation that all embryonic macrophage populations (yolk sac-derived, fetal liver-derived, and bone marrow-derived) can independently colonize an empty alveolar niche and differentiate into functional AM suggests that the lung tissue produces maturation and development signals that can turn embryonic progenitors into differentiated AM. In this setting, the production of granulocyte macrophage colony-stimulating factor (GM-CSF) by the lung stroma, specifically by the alveolar epithelial type 2 cells [14], is vital since it induces the expression of the transcription factor peroxisome proliferator-activated receptor gamma (PPARG), a transcription factor essential for the differentiation and perinatal development of AM [15]. The role of GM-CSF is also supported by the demonstration that, in a situation of competition between progenitors, fetal liver progenitors, which have the highest affinity and avidity for this growth factor, become the main contributor to the replenishment of the alveolar niche [8]. As for the differentiation and maintenance of the AM population, the transforming growth factor beta (TGFB) appeared to be crucial in the alveolar niche [16]. The roles and mechanisms of tissue imprinting in AM differentiation and maintenance programs were recently demonstrated. Indeed, while differentiated immune cells usually lose their plasticity, differentiated peritoneal macrophages can acquire the main features of AM after in vivo adoptive transfer into the lungs [17]. This result demonstrated the high plasticity of differentiated macrophages and the role of the tissue microenvironment in both the induction and the maintenance of their functional programming by shaping the chromatin landscape. In summary, even during homeostasis, the maintenance of the functions and identity of AM relies on their local self-proliferation with minimal participation from monocytes, and their constant adaptation to the messages received from the local microenvironment [1,8,9].

## 4. The Role of Alveolar Macrophages to Maintain Lung Homeostasis

Lungs are not sterile but are continuously exposed to foreign molecules [18] and colonized by a diverse microbiome. The balance between immune response to eliminate these foreign bodies and tolerance is highly regulated in the lungs, with tolerance favored during homeostasis [19,20]. This is particularly crucial since lung inflammation causes thickening of alveoli walls, jeopardizes gas exchange, and induces life-threatening respiratory failure. Nevertheless, tolerance of virulent pathogens can lead to invasive infections, and it is thus necessary to finely tune the balance between tolerance and immune response.

While the historical theory was that AM induce lung immune tolerance, recent studies have also underlined their central role in maintaining epithelium integrity [21]. AM continuously patrol alveoli to clean the alveolar spaces by phagocytizing inhaled bacteria before they can initiate harmful lung inflammation [22]. AM, which can present antigen but lack co-stimulation molecules, inactivate the CD8+ T cells localized in the airways [23,24]. In humans, AM closely interact with resident memory T cells, which are central to optimize immune response against inhaled pathogens [25].

**Figure 1:**
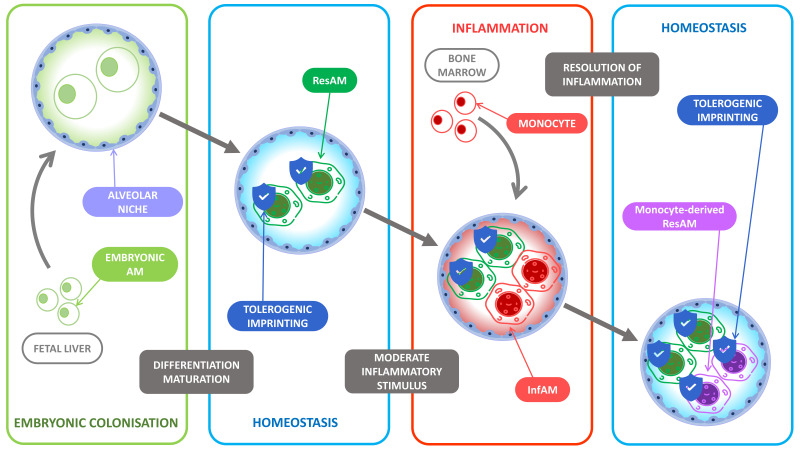
Tolerogenic imprinting throughout inflammation. At birth, the alveolar niche is colonized by fetal liver-derived monocytes. The lung microenvironment then favors their differentiation into resident alveolar macrophages (ResAM) and induces an unalterable tolerogenic imprinting. During inflammation, the main inflammatory actors are the recruited bone marrow-derived monocytes that differentiate into monocyte-derived alveolar macrophages (InfAM) under the pressure of the proinflammatory microenvironment while the ResAM display poor inflammatory functions as most of their tolerogenic imprinting remains. At the resolution of inflammation, the recruited InfAM become monocyte-derived ResAM and undergo the tolerogenic pressure of the microenvironment and display in turn a tolerogenic imprinting to limit further inflammatory response.

All these tolerogenic functions of the AM can be reversed during pneumonia, as we will discuss later, suggesting a microenvironment pressure that remains to be clarified. On the whole-body scale, tissue macrophages differentiation depends on signals given by the macrophage niche [26] but as far as the intrinsic macrophage genetic program is concerned, it is hypothesized that the microenvironment represses proinflammatory genes and promotes an immune tolerance which does make sense in an evolutionary perspective: the new macrophages that replace the dead ones should be set in a tolerogenic mode to prevent unnecessary and potentially deleterious inflammation [27], and this microenvironment pressure applies on AM independently of their ontogeny [17,28,29,30] (Figure 1).

**Figure 2:**
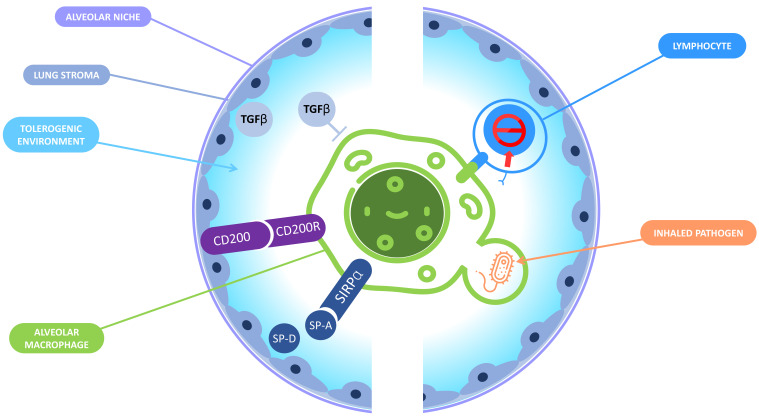
Immune tolerance in the alveolar niche. In the alveolar niche, macrophages and the lung stroma interact together to promote a tolerogenic environment and prevent a deleterious inflammation that could jeopardize the lungs functions. On the one hand, the lung microenvironment induces tolerance in AM: the binding of CD200R expressed by the AM and CD200 expressed by the lung stroma inhibits the macrophages response; lung epithelial cells produce surfactant proteins A and D that can interact with signal-regulatory protein (SIRPA) receptor leading to phagocytosis inhibition; TGB-β is also produced by lung epithelial cells and is responsible for the inactivation of the AM. On the other hand, AM display tolerogenic functions including the inactivation of CD8+ T lymphocytes and the «silent» phagocytosis of inhaled pathogens.

Several mediators from the microenvironment participate to the tolerogenic programming of ResAM. First, the metabolic adaptation of ResAM to a local environment poor in nutriments impacts their ability to engulf CD47+ tumor cells and to phagocytize extracellular bodies [31]. The stimulation of CD200R upon the production of CD200 by lung epithelial cells negatively regulates the AM response to bacteria [32]. Epithelial cells, as well as AM themselves, also produce TGFB that limits the activation of AM and promotes their self-maintenance [16,33]. Another element promoting the tolerance of AM is the surfactant proteins A and D, which are collagen-containing C-type lectins produced by epithelial cells which interact with the signal-regulatory protein alpha (SIRPA) receptor expressed on AM [34]. Surfactant proteins stimulation of SIRPA inhibits the phagocytosis of extracellular bacteria, while the recognition by SIRPA of CD47 precludes the phagocytosis of cells from the host [35,36]. In summary, the lung microenvironment drives ResAM toward tolerogenic functions, which are critical to prevent potentially detrimental lung inflammation (Figure 2).

## 5. The Evolution of Alveolar Macrophage Niche Complexity during Infection and Inflammation

**Figure 3:**
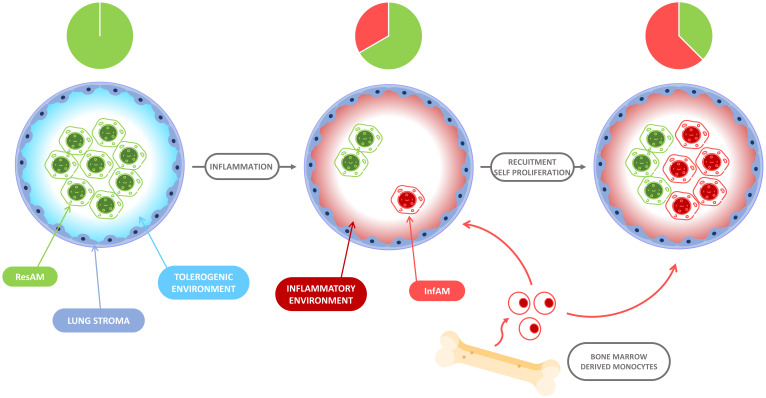
Evolution of the alveolar niche after inflammation. During homeostasis, the alveolar niche contains ResAM. After lung inflammation, a variable proportion of the alveolar niche is depleted and bone marrow-derived monocytes are recruited to fill the space. On the one hand, ResAM replenish the niche by self-proliferation and, on the other hand, the recruited monocytes differentiate into AM in a proinflammatory environment: InfAM. The intensity of the inflammation process dictates the proportions of ResAM and InfAM within the alveolar niche.

**Figure 4:**
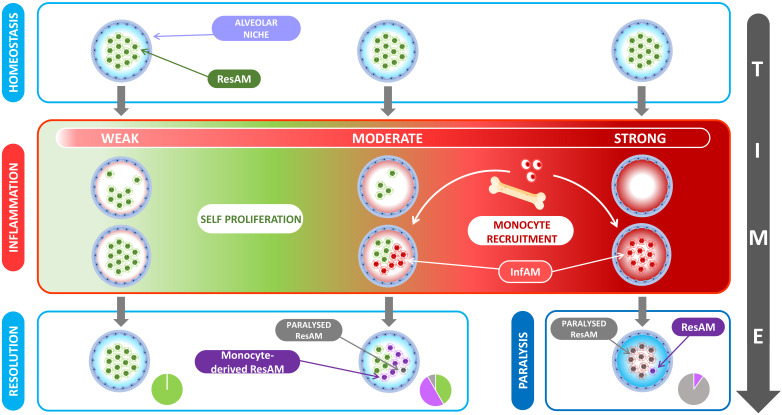
Role of the stimulus intensity and AM niche organization. During a weak intensity inflammation, the ResAM depletion is scarce and the remaining ResAM can repopulate the niche by self-proliferation without the contribution of circulating monocytes. During a moderate intensity inflammation, the ResAM depletion is more important and the self-proliferation is not enough. Circulating monocytes are recruited and differentiate in an inflammatory context into InfAM. At the resolution of the inflammation, the niche is enriched with a new population of ResAM originated from the recruited monocytes and some monocyte-derived AM display a paralyzed phenotype. During a strong intensity inflammation, the alveolar niche is totally depleted and no ResAM are left to self-proliferate. The recruited monocytes differentiate into InfAM and constitute the main population of the niche. At the resolution of the inflammation, the AM are mostly paralyzed as the microenvironment promotes an excessive tolerance leading to a global immune suppression. The intensity of the inflammation is responsible for proportions changes of different cell subtypes in the alveolar niche.

While the AM pool remains relatively stable with minimal contribution from circulating monocytes in specific-pathogen-free mice models, it is unlikely the case in humans when lungs are regularly exposed to foreign bodies and pathogens. Indeed, recent studies have demonstrated continuous recycling and diversification of the AM origins and functions throughout life according to the history of lung injuries (Figure 3). The nature and the severity of the lung aggressions can cause various ResAM depletion depths (from no depletion to complete depletion) (Figure 4).

While in the absence of lung inflammation, the contribution of circulating monocytes to the renewal of the ResAM pool is limited, as demonstrated in different mice models [1], they can fully colonize the alveolar niche when the depletion of ResAM is complete such as after irradiation [8,37], lipopolysaccharide-induced acute lung injuries [38,39], or during chronic viral lung infection [40]. In the case of less severe lung inflammation with partial depletion of ResAM, resident and recruited AM can coexist in the lungs. For instance, Aegerter et al. [41] reported that one month after an acute respiratory influenza A infection, a subset of AM originated from recruited monocytes remained in the alveoli. These monocyte-derived AM were not distinguishable from the initial ResAM on a phenotypic level, but significant transcriptomic and epigenetic differences remained apparent. While the phenotype of ResAM and monocyte-derived AM became very similar after a few weeks of lung co-localization, functions and epigenetic regulations of the recruited AM did not completely recapitulate the status of ResAM described in homeostasis.

As proposed by Guilliams et al. [27], we have defined AM which colonize the lungs during embryonic life and homeostasis, as ResAM, and those that derived from circulating monocytes and colonize the lung following tissue injuries, as InfAM. The mechanisms regulating the proportions of ResAM and InfAM after replenishment and the persistence of functional differences between these cell populations remain incompletely understood. The nature of the depleting agent (virus, bacteria, toxic agent, irradiation), the degree of the niche depletion, and the magnitude and duration of the lung inflammation likely regulate the competition between ResAM and InfAM and their reprogramming. Several questions remain to be addressed: do ResAM and InfAM interact together? Are InfAM able to definitively settle in the anatomic niche and share the space with ResAM in a scenario of incomplete ResAM depletion? What are the critical mediators from the cellular microenvironment involved in the specific training of ResAM and InfAM? Does the respiratory microbiota participates in the ResAM and InfAM programming?

## 6. Differences between ResAM and InfAM during Inflammation

During lung inflammation, ResAM and InfAM coexist and collaborate to restore homeostasis by eliminating the cause of the danger signals on the one hand and by dampening the inflammation on the other hand. However, ResAM and InfAM likely have different functions during healing. A few studies have distinctly compared ResAM and InfAM populations since most of the former murine models of respiratory infections have not distinguished these two subtypes because of their identical phenotypes or complete deletion of ResAM. New tools are now available and make it possible to decipher the role of each cell subtype selectively. Mice models of constitutional deletion of macrophages usually do not eliminate the recruitment of InfAM, while it is possible to prevent it by blocking the CCL2-CCR2 axis [42]. It is also possible to induce a chimerism by adoptive cell transfer or by generating bone marrow chimeras where ResAM and InfAM do not express the same CD45 antigens and can thus be tracked in vivo.

Mice constitutively depleted of ResAM (*Csf*^−/−^ and *Cd11c*^Cre^/*Pparg*^fl/fl^ mice) [43] have higher morbidity and mortality rates during a respiratory influenza virus infection despite the functional recruitment of InfAM and normal adaptive immunity. Mice depleted of ResAM by intrapulmonary instillation of clodronate liposome or liposomal dichloromethylene-bisphosphonate had worse outcomes than the control mice and displayed higher levels of proinflammatory cytokines and higher accumulation of apoptotic and necrotic neutrophils during *Streptococcus pneumoniae* pneumonia [44]. The depletion of ResAM increased the systemic diffusion of pathogens notably during *Brucella abortus* infection [45]. ResAM also decrease neutrophil-mediated epithelial damages and restore homeostasis by cloaking over tissue microlesions [46]. ResAM can also regulate the functions of other immune cells, notably through the production of type I interferon secondary to respiratory viral infection [47]. These results demonstrate that ResAM play a significant role in lung homeostasis by preventing the initiation of immune response and dampening the inflammation during sepsis.

InfAM are rapidly imprinted by the inflammatory local microenvironment making them suitable for proinflammatory and immunogenic functions. During the early days of response to *Legionella pneumophila* infection, InfAM are a significant source of interleukin 12 (IL-12), inducing interferon gamma (IFNG) production by natural killer cells and T cells [48]. Using *Ccr2*^−/−^ mice to limit the recruitment of monocytes and thus the formation of InfAM, Aegerter et al. [41] demonstrated that InfAM has a high capacity of IL-6 production during influenza viral infection, thus increasing the resistance to secondary pneumonia by *Streptococcus pneumoniae*. The differences in inflammatory cytokines production between ResAM and InfAM are regulated at an epigenetic level, as demonstrated by the open chromatin region of *Il6* and high cytokine signaling immune system function in transcriptomic analyses. All these modifications are mainly driven by the different ways the tissue imprints ResAM and monocytes maturing into macrophages. Some research teams have investigated the role of intrinsic programming of monocytes, i.e., the conditioning of these cells in the bone marrow. Askenase et al. [49] found that bone marrow monocytes acquired a regulatory phenotype during a digestive infection before migrating to the infected site. Similarly, but without bringing the actual evidence that these modifications occurred inside the bone marrow, monocytes recruited after a viral murid herpesvirus 4 infection [40] or respiratory syncytial virus [50] acquired regulatory and antiviral functions. Once in the alveoli, the InfAM start expressing *Csf1* and *Pdgfa* and survive via an autocrine macrophage colony-stimulating factor (M-CSF) stimulation thus becoming monocyte-derived ResAM [51]. Some studies have also reported that monocytes can differentiate into short-lived macrophages, which disappear at the resolution of inflammation [1]. The reasons why these transient AM do not become ResAM, and the differences of role between monocyte-derived transient AM and monocyte-derived ResAM remain largely unknown [27].

**Figure 5:**
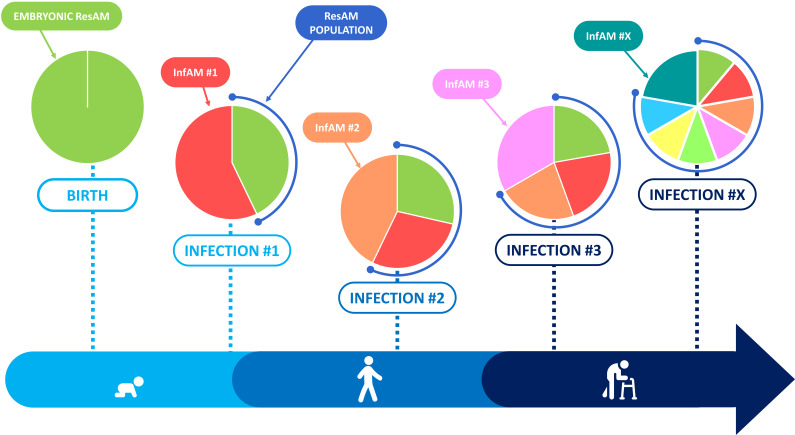
The complexity of human alveolar niche. At birth, the alveolar niche contains embryonic ResAM but during a lung inflammation, a wave of InfAM arrives and they become at the resolution of inflammation, the new monocyte-derived ResAM. Every inflammation generates a new InfAM recruitment wave and at the end, contributes to the heterogeneity of the ResAM population throughout life.

Future research will have to thoroughly investigate the division of labor between embryonic- and monocyte-derived ResAM during the very early days of pulmonary infections. This will likely help to define sub-phenotypes of the inflammatory course associated with pneumonia outcomes in humans. However, the study of human respiratory samples raises another challenge: if the distinction between ResAM and InfAM can likely be performed via high-throughput cytometry, it appears particularly challenging to distinguish the InfAM that the ongoing infection has recruited from the ResAM originating from trained monocytes that are recruited during previous inflammatory episodes [27] (Figure 5).

## 7. Tissue Repair and Fibrosis

During the resolution of the inflammation, the macrophage populations have emerged as a serious candidate in the pathophysiology of fibrosis [52,53,54]. The mechanisms of fibrosis rely on the activation of fibroblasts in a profibrotic environment [55]. The bleomycin-treated mice model is considered the main experimental pulmonary fibrosis model because the mechanisms are very similar with what is observed in humans [55,56]. Thanks to this historical model, a link was made between lung fibrosis and bone marrow progenitors [56], later identified as monocyte-derived AM [57]. A decrease in the severity of the bleomycin-induced fibrosis and its clinical burden among mice was reported without the capacity of recruiting monocytes in the lungs [52,53]. More recently, as the distinction between ResAM and InfAM became easier, it was found that ResAM are not deeply implicated in the bleomycin-induced fibrosis, whereas InfAM seemed to play a central role [58]. Indeed, InfAM display a profibrotic transcriptomic signature notably characterized by a high production of platelet derived growth factor receptor alpha (PDGFA), the main fibroblast activator [57]. Interestingly, the expression of profibrotic genes by InfAM was induced locally after the recruitment into the lung tissue, and then decreased over time [58]. As the level of profibrotic genes expression decreased, the macrophage residency signature increased over time. One year after bleomycin treatment, the differences between embryonic ResAM and monocyte-derived ResAM became transcriptionally very thin and phenotypically almost impossible. If several hypotheses were proposed among a weakening of the environmental signals and interactions between embryonic and monocyte-derived ResAM, the mechanisms of this time-dependent genetic program remain largely unelucidated.

The investigation of the pulmonary fibrosis has strengthened the observations made during the acute inflammation phase: the embryonic ResAM seem to hold the central role in preventing every deleterious excess (inflammation or fibrosis). The regulation of the functions of InfAM also appears critical since excessive and uncontrolled fibrosis can lead to chronic respiratory failure and long-term sequelae in humans [59]. In this sense, understanding the regulation of the fibrosis program duration could bring new insight into a therapeutic approach.

Future research should assess the long-term fate of the monocyte-derived ResAM. Some research teams state that ResAM and InfAM become indistinguishable [28], while others still find subtle transcriptomic differences or a different behavior during secondary aggressions [41]. It appears that monocyte-derived ResAM can keep a memory of their recruitment period known as trained immunity. Interestingly, this supposes that each resolved pulmonary inflammation enriches the macrophage niche to be prepared for a potential next round of inflammation [27]. We could further hypothesize that the innate memory could complete the adaptive memory since we tend to observe that the action of the AM limits the adaptive immunity, probably in an evolutionary lung-protective way [60].

## 8. The Immunologic Scar Left over by Inflammation: Friend of Foe?

After the cure from primary infection or acute inflammation, the quality of the innate immune surveillance is modified for months. Macrophages and dendritic cells are the most studied cells involved in this concept of innate immune memory, which is presented as a primitive form of host defense adaptation to the environment [61]. The pre-exposure to pathogens induces either the phenomena of trained immunity characterized by increased metabolic response and cytokine production during restimulation [62], or the phenomena of the LPS-induced immunological tolerance [63]. Training and tolerance programming are characterized by profound chromatin structure rearrangements, such as histone methylation (H3K4me3) and acetylation (H3K27ac), that modulate the chromatin accessibility and activate or repress gene transcription [62,64]. Profound metabolic modifications, notably the itaconate pathway, also regulate innate immune tolerance and trained immunity [65]. In vivo, the reprogramming can differ according to the tissue and primary stimulus type and intensity. After primary bacterial pneumonia, conventional dendritic cells lose their ability to present newly encountered antigens and produce inflammatory cytokines after bacterial pneumonia, but gain TGFB dependent tolerogenic functions, resulting in local accumulation of FoxP3 regulatory T cells and higher susceptibility to infections [66]. After respiratory viral infection, the training of ResAM increases the membrane expression of major histocompatibility complex II (MHC-II), the glycolytic activity, and the proinflammatory cytokines production but decreases their phagocytic ability [67]. These features consist of a “defense-ready” genetic signature and an increased resistance to secondary pneumonia [68]. Similarly, in the bleomycin-induced lung fibrosis model, monocyte-derived AM recruited after the first exposure to bleomycin could react more efficiently and more intensely to second bleomycin aggression, which was not the case if the second aggression was of a viral type [69].

These modifications are primed by inflammatory stimuli then self-maintain when the stimulus is gone. ResAM have a slower turnover than dendritic cells, yet the life expectancy of mononuclear phagocytic cells usually does not exceed a few weeks. When investigating in vivo the mechanisms of trained innate immunity, it is important to discriminate the initiation and the maintenance programs. Supporting this message, we have reported that the phagocytosis capacity of trained ResAM was decreased after *Escherichia coli* pneumonia, through a SIRPA dependent mechanism [67]. However, while the functional reprogramming of ResAM was not observed in *Sirpa*^−/−^ mice, SIRPA deficient ResAM still became trained after an adoptive transfer into wild-type mice cured from a primary infection [67]. While this series of experiments showed that the training of ResAM was induced by the local microenvironment, the intrinsic modifications of ResAM induced at the time of inflammation were also involved. For instance, the production of IFNG by CD8 T cells, which only lasts a few days during viral pneumonia, was shown to prime ResAM which become memory ResAM with a “defense ready” signature for months after the cure from pneumonia.

Several other mechanisms are proposed to explain the maintenance of functional reprogramming: modifications of the bone marrow precursors which gave rise to monocyte-derived ResAM, the replacement of embryonic ResAM by monocyte-derived cells, and the alterations of the tissue imprinting. Reinforcing the first one, modifications of bone-marrow precursors at the time of sepsis or anti-tubercular bacillus of the Calmette and Guérin (BCG) vaccination, a historical tool to induce training in humans, are described [70]. Three months after vaccination with BCG, the transcriptomic remodeling of hematopoietic stem and progenitor cells collected in healthy humans was passed over to peripheral CD14+ monocytes, displaying an activated transcriptional signature. Even in the absence of direct effect of sepsis on bone marrow monocyte progenitors, activated natural killer cells can directly prime monocytes for tolerogenic functions before their bone marrow egress [49]. This mechanism is also supported by the demonstration that the phagocytosis capacity of circulating monocytes was reduced for months following a severe systemic inflammation (sepsis, brain injury) [67].

In some experimental conditions, bacterial and adenovirus infections can cause a severe depletion of ResAM, making it possible for the recruited monocytes to develop into monocyte-derived ResAM and settle in the depleted anatomical niche [40,71,72,73]. As described above, these newly formed cells become phenotypically closed to ResAM but functionally distinct. For instance, after viral pneumonia, the regulatory monocyte-derived AM can dampen the symptoms of house dust mite-induced asthma [40] or increase protection against *Streptococcus pneumoniae* secondary infection through high IL-6 production [41].

Finally, as described above, macrophages are highly plastic cells, and several teams have demonstrated that ResAM and dendritic cells can be locally reprogrammed by modifications in the cellular or microbial environment [67,68,74]. Notably, acute inflammation increases the local concentration of CD4 regulatory FoxP3 cells and memory T cells which have the theoretical potential to regulate ResAM.

While the modulation of ResAM training is a promising therapeutic target to reduce the risks of hospital-acquired pneumonia and death after critical illness [75,76,77], all these mechanisms likely combine, making it difficult to therapeutically annihilate the functional reprogramming of ResAM in humans. Moreover, the trained immunity response, that varies in intensity and effects, according to the nature and the localization of the stimulus, can be beneficial or detrimental to the host [78]. We proposed that a better comprehension of the role of the AM niche complexity in the susceptibility to secondary medical conditions during trained immunity will help to design new biomarkers and therapeutic approaches. Aegerter et al. [41] hypothesized the very plausible notion that the initial stimulus intensity directly impacts the macrophage niche’s fate: low-intensity aggression would induce protective trained immunity, whereas high-intensity aggression (potentially lethal) would induce a deleterious immune modulation from chronic inflammation to immune paralysis. Moreover, the type of pathogens, for instance viral vs. bacterial pneumonia, areassociated with different lymphocytes and monocytes response during the initiation of the reprogramming of ResAM [79]. Van der Poll et al. [80] further found that long-term innate immune cell reprogramming impacted cytokine production and could be linked to post septic immune paralysis. More discrete data even suggested a link between post-critical immune modulation and long-term risk of cancer [81]. The study of the AM niche opens a vast and ever-growing field of applications. New insights into the post inflammatory immune modulation via trained immunity could lead to innovative immunotherapies which could safely induce protective trained immunity in order to prevent or treat respiratory infections by stimulating the critically ill patients’ immunity suffering from immune paralysis [82] or by inducing a regulatory trained immunity in the context of COVID-19 SARS-CoV-2 infection [83].

## 9. Conclusions

Lungs, which are continuously exposed to inhaled particles and pathogens, need to develop a robust tolerogenic environment preventing inflammation-induced wall thickening during homeostasis, yet to be able to switch to a proinflammatory response in cases of virulent pathogens inducing pneumonia. The complementary actions of tolerogenic ResAM, which mainly hide danger from immune systems and dampen inflammation, and proinflammatory InfAM, which are recruited in case of tissue injury and decrease pathogen burden at the price of immunopathology, thus appear a perfect match [45,84,85]. The study of the alveolar niche’s dynamics brings light to the pathophysiology of pulmonary inflammation. The lung microenvironment plays a critical role in macrophages functions and their genetic imprinting. The tolerogenic pressure it applies in homeostasis protects the lung tissue integrity. However, in the context of lung inflammation, the microenvironment seems to allow a temporary inflammatory signature on the recruited monocytes to fight the aggression and, only then, undergo the tolerogenic pressure. This mechanism can be self-destructive in a context of a high-intensity inflammation, as it is the case among critically ill patients who are susceptible to hospital-acquired pneumoniae because of an excessive immune tolerance that leads to immune paralysis following an initial inflammation. Deciphering the mechanisms of the impact of the lung microenvironment on AM would allow to therapeutically modulate lung immunity in situations where the latter fails.
